# Impact of remote patient monitoring on clinical outcomes: an updated meta-analysis of randomized controlled trials

**DOI:** 10.1038/s41746-017-0002-4

**Published:** 2018-01-15

**Authors:** Benjamin Noah, Michelle S. Keller, Sasan Mosadeghi, Libby Stein, Sunny Johl, Sean Delshad, Vartan C. Tashjian, Daniel Lew, James T. Kwan, Alma Jusufagic, Brennan M. R. Spiegel

**Affiliations:** 1 Division of Health Services Research, Cedars-Sinai Medical Center, Los Angeles, CA USA; 2 Cedars-Sinai Center for Outcomes Research and Education (CS-CORE), Los Angeles, CA USA; 3 Department of Health Policy and Management, UCLA Fielding School of Public Health, Los Angeles, CA USA; 4 Department of Medicine, University of Arizona, College of Medicine Tucson, Tucson, AZ USA; 5 Cedars-Sinai Medical Center, Los Angeles, CA USA; 6 American Journal of Gastroenterology, Bethesda, USA

**Keywords:** Disease prevention, Weight management, Health services

## Abstract

Despite growing interest in remote patient monitoring, limited evidence exists to substantiate claims of its ability to improve outcomes. Our aim was to evaluate randomized controlled trials (RCTs) that assess the effects of using wearable biosensors (e.g. activity trackers) for remote patient monitoring on clinical outcomes. We expanded upon prior reviews by assessing effectiveness across indications and presenting quantitative summary data. We searched for articles from January 2000 to October 2016 in PubMed, reviewed 4,348 titles, selected 777 for abstract review, and 64 for full text review. A total of 27 RCTs from 13 different countries focused on a range of clinical outcomes and were retained for final analysis; of these, we identified 16 high-quality studies. We estimated a difference-in-differences random effects meta-analysis on select outcomes. We weighted the studies by sample size and used 95% confidence intervals (CI) around point estimates. Difference-in-difference point estimation revealed no statistically significant impact of remote patient monitoring on any of six reported clinical outcomes, including body mass index (−0.73; 95% CI: −1.84, 0.38), weight (−1.29; −3.06, 0.48), waist circumference (−2.41; −5.16, 0.34), body fat percentage (0.11; −1.56, 1.34), systolic blood pressure (−2.62; −5.31, 0.06), and diastolic blood pressure (−0.99; −2.73, 0.74). Studies were highly heterogeneous in their design, device type, and outcomes. Interventions based on health behavior models and personalized coaching were most successful. We found substantial gaps in the evidence base that should be considered before implementation of remote patient monitoring in the clinical setting.

## Introduction

Wearable biosensors are non-invasive devices used to acquire, transmit, process, store, and retrieve health-related data.^1^ Biosensors have been integrated into a variety of platforms, including watches, wristbands, skin patches, shoes, belts, textiles, and smartphones.^2,3^ Patients have the option to share data obtained by biosensors with their providers or social networks to support clinical treatment decisions and disease self-management.^4^

The ability of wearable biosensors to passively capture and track continuous health data gives promise to the field of health informatics, which has recently become an area of interest for its potential to advance precision medicine.^1^ The concept of leveraging technological innovations to enhance care delivery has many names in the healthcare lexicon. The terms digital health, mobile health, mHealth, wireless health, Health 2.0, eHealth, quantified self, self-tracking, telehealth, telemedicine, precision medicine, personalized medicine, and connected health are among those that are often used synonymously.^5^ A 2005 systematic review uncovered over 50 unique and disparate definitions for the term e-health in the literature.^6^ A similar 2007 study found 104 individual definitions for the term telemedicine.^7^ For the purpose of this study, we employ the term remote patient monitoring (RPM) and define it as the use of a non-invasive, wearable device that automatically transmits data to a web portal or mobile app for patient self-monitoring and/or health provider assessment and clinical decision-making.

The literature on RPM reveals enthusiasm over its promises to improve patient outcomes, reduce healthcare utilization, decrease costs, provide abundant data for research, and increase physician satisfaction.^2,3,8^ Non-invasive biosensors that allow for RPM offer patients and clinicians real-time data that has the potential to improve the timeliness of care, boost treatment adherence, and drive improved health outcomes.^4,9^ The passive gathering of data may also permit clinicians to focus their efforts on diagnosing, educating, and treating patients, theoretically improving productivity and efficiency of the care provided.^8^ However, despite anecdotal reports of RPM efficacy and growing interest in these new health technologies by researchers, providers, and patients alike, little empirical evidence exists to substantiate claims of its ability to improve clinical outcomes, and our research indicates many patients are not yet interested in or willing to share RPM data with their physicians.^4^ A recently published systematic review by Vegesna et al. summarized the state of RPM but provided only a qualitative overview of the literature.^10^ In this review, we provide a quantitative analysis of RPM studies to provide clinicians, patients, and health system leaders with a clear view of the effectiveness of RPM on clinical outcomes. Specifically, our study questions were as follows: How effective are RPM devices and associated interventions in changing important clinical outcomes of interest to patients and their clinicians? Which elements of RPM interventions lead to a higher likelihood of success in affecting clinically meaningful outcomes?

We sought to identify randomized controlled trials (RCTs) that assess the effects of using non-invasive, wearable biosensors for RPM on clinical outcomes. Understanding precisely in which contexts biosensors can improve health outcomes is important in guiding research pathways and increasing the effectiveness and quality of care.

## Results

### Study selection and data collection

**Fig. 1:**
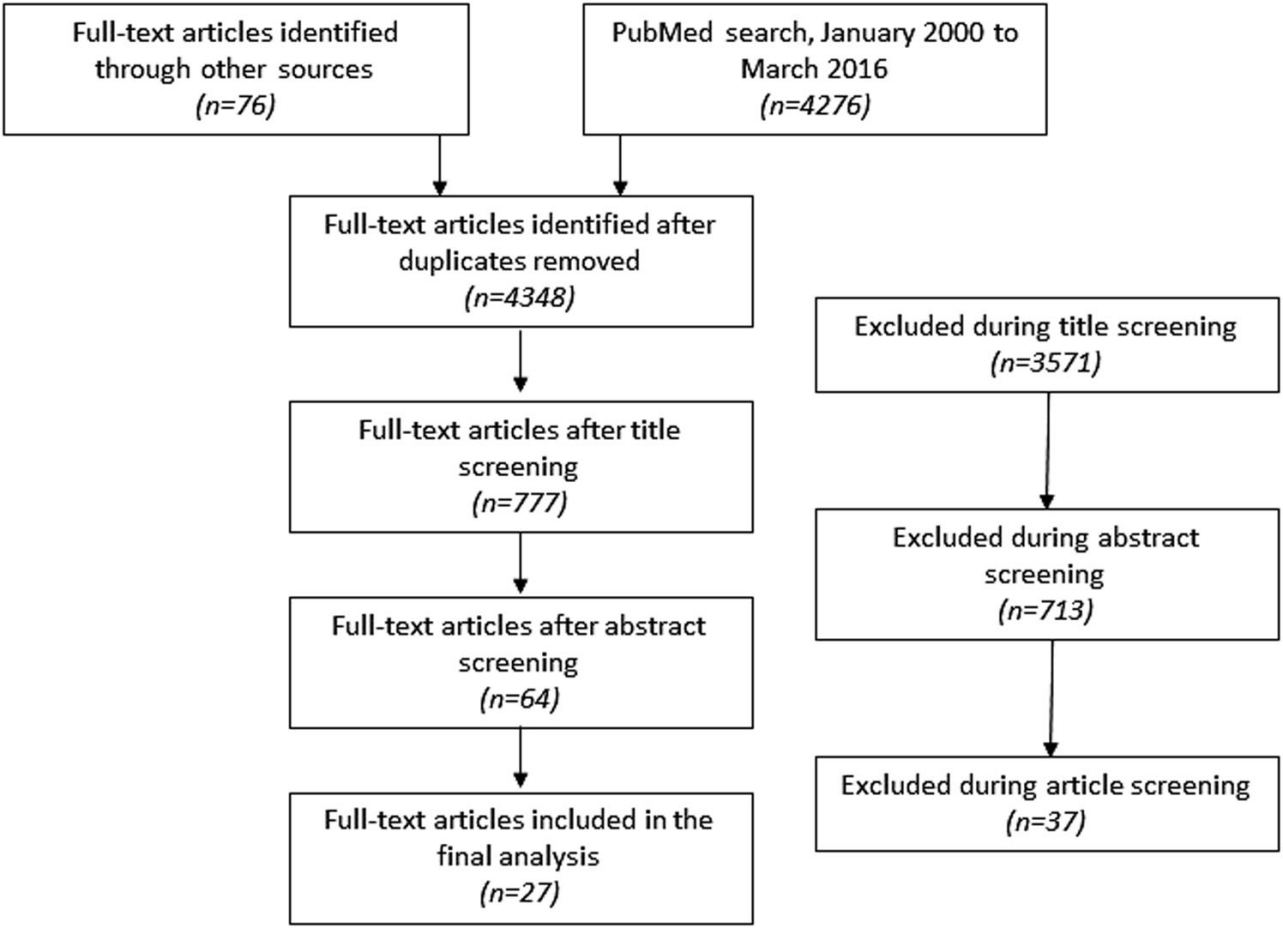
PRISMA flow diagram of the process used in study selection

We identified 4348 titles for review (Fig. 1). Of these, we selected 777 for abstract review and 64 for full-text review. A total of 27 studies were retained for final analysis.^11–35^ All studies were RCTs published in peer-reviewed journals.

### Quantitative identifiers

#### Study details

The 27 studies analyzed had an average study duration of 7.8 months. The study periods ranged from 7 days to 29 months (Table 1). The average sample size was 239 patients and ranged from 40 to 1437 patients. Sixteen studies were determined to be of high quality, with a Jadad score equal to 3. Eleven studies were determined to be of low quality. The mean Jadad score for all 27 studies identified in this review was 2.44 (Table 1). Since it is often not feasible to double-blind interventions with wearable devices, the maximum Jadad score in these trials was 3.

**Table 1:** Remote patient monitoring systematic review study

First author, year	Study duration	Sample size	Percent male (%)	Mean age	High quality study?^a^
Scalvini, 2005	7 days	310	24	52.5	No
Dansky, 2008	4 months	284	N/A	77	No
Woodend, 2008	15 months	249	75.1	63.6	No
Tan, 2010	2 weeks	120	40.6	48	No
Shuger, 2011	9 months	197	18.3	46.9	Yes
Chau, 2012	2 months	40	97.5	72.9	Yes
Dinesen, 2012	4 months	105	N/A	68	Yes
Fox, 2012	3 months	75	80	53.5	No
Logan, 2012	12 months	105	55.7	49	Yes
Ryan, 2012	6 months	288	37.4	49	No
De San Miguel, 2013	6 months	80	48.5	72.5	Yes
Greene, 2013	6 months	349	21.1	N/A	No
Lee, 2013	3 months	55	80.2	56.1	No
Pedone, 2013	9 months	99	67.5	74.7	Yes
Wijsman, 2013	3 months	226	59.2	64.8	Yes
Luley, 2014	12 months	178	58.7	50.3	No
Piga, 2014	3 months	40	2.5	56.9	Yes
Dorsch, 2015	15 months	135	59.3	62.3	Yes
Kent, 2015	14.5 months	112	40.8	43.3	Yes
Kim, 2015	29 months	374	58	57.1	Yes
Pedone, 2015	6 months	90	38.9	79.8	No
Wang, 2015	1.5 months	67	8.9	48.2	Yes
Bloss, 2016	6 months	130	44	55.5	No
Ginis, 2016	2.5 months	38	N/A	N/A	Yes
Ong, 2016	3 months	1437	53.1	73.5	Yes
Finkelstein, 2016	12 months	800	46.3	35.5	Yes
Jakicic, 2016	24 months	470	22.8	N/A	Yes

^a^ A high-quality study is a study with a Jadad score ≥ 3 (5-point scale) (15)

#### Study outcomes

Eleven studies examined patient populations with cardiovascular disease, including heart failure, arrhythmias, and hypertension. Six studies evaluated patients with pulmonary diseases, including emphysema, asthma, and sleep apnea. Six trials examined overweight or obese patients or tested interventions aimed at increasing physical activity to prevent weight gain. The remaining studies focused on chronic pain, stroke, and Parkinson’s disease (Table 2).

**Table 2:** RPM study categories and device types

First author, year	Country	Disease state	Device	Control	Primary outcomes (− / NS /+)	Care provider involved?	Meta-analysis measures	Population type
Lee, 2013	Korea	Acute coronary syndrome	Wireless electrocardiography device to check heart rate during exercise	Ordinary medical therapy, diet control, and exercise	Exercise capacity (+)	Yes	None	Adults with acute coronary syndrome who recently underwent a successful percutaneous coronary intervention
Tan, 2010	Singapore	Cardiac arrhythmia	Internet-based ambulatory ECG monitoring device	Transtelephonic event recorder	Diagnostic yield (NS)	Yes	None	Patients from the National Heart Centre, Singapore
Chau, 2012	China	COPD	Pulse oximeter, respiratory rate sensor	No devices, education only	Pulmonary function (NS), hospital readmissions (NS), ER usage (NS), HRQL (NS)	Yes	None	Adults 60 years or older with moderate-to-severe COPD
Dinesen, 2012	Denmark	COPD	Telehealth monitor that collected blood pressure, pulse, weight, and lung function	Instructions on home exercises only	Admission rates (+) and mean cost of admissions costs (NS)	Yes	None	Adults 18 years or older with severe-to-very severe COPD
De San Miguel, 2013	Australia	COPD	Device that measures blood pressure, weight, temperature, pulse, and oxygen saturation	Education only	Healthcare utilization (NS)	Yes	None	Adults with COPD receiving domiciliary oxygen
Pedone, 2013	Italy	COPD	Pulse oximeter; wristband that measured heart rate, physical activity, and temperature	Standard care	Number of exacerbations and hospitalizations (+)	Yes	None	Adults 65 or older with COPD in GOLD stages II and III
Dansky, 2008	USA	Heart failure	Blood pressure, pulse, and weight monitoring system; digital stethoscope	Routine home visits only	Hospitalizations and ED visits (NS)	Yes	None	Patients with a primary or secondary diagnosis of heart failure
Pedone, 2015	Italy	Heart failure	Telemonitoring system measuring blood pressure, heart rate, weight, and oxygen saturation	Education only	Hospital admissions and mortality (+)	Yes	None	Adults 65 years or older with heart failure
Ong, 2016	USA	Heart failure	Telemonitoring system measuring blood pressure, heart rate, and weight	Education only	Readmission within 180 days after discharge (NS)	Yes	None	Adults 50 years or older with active treatment for heart failure
Woodend, 2008	Canada	Heart failure and angina	Electronic weight scale, blood pressure monitor, and electrocardiogram	Usual care for patients discharged with HF or angina	Hospital readmissions (+), days spent in hospital (+)	Yes	None	Adults with symptomatic heart failure or agina
Luley, 2014	Germany	Metabolic syndrome	Accelerometer tracking physical activity	Education only	Weight Loss (+)	Yes	Body mass index, weight, waist circumference, systolic blood pressure, diastolic blood pressure	Adults aged 30–60 with a diagnosis of metabolic syndrome
Scalvini, 2005	Italy	Palpitations, cardiac arrhythmias	At home trans-telephonic event recorder	At home Holter monitoring	Number of total diagnoses (+) and total costs (+)	Yes	None	Adults with intermittent palpitations
Dorsch, 2015	USA	Stroke	Accelerometer with feedback from data	Accelerometer without feedback	Total daily walking time (NS) and timed 15 m walk (NS)	Yes	None	Patients with chronic hemiparetic stroke
Logan, 2012	Canada	Hypertension	Blood pressure with smartphone application	Home blood pressure monitor without transmission of data	Daytime ambulatory systolic blood pressure (+)	Yes	Systolic blood pressure, diastolic blood pressure	Adults older than 30 years with diabetes mellitus
Kim, 2015	Korea	Hypertension	Blood pressure monitor with remote monitoring	Blood Pressure measurement without remote monitoring	Sitting systolic blood pressure (NS)	Yes	Systolic blood pressure, diastolic blood pressure	Patients 20 years or older with hypertension
Bloss, 2016	USA	Hypertension, diabetes, cardiac arrhythmias	Blood pressure monitor and mobile ECG	Education and website for disease management	Total health insurance claims and visits to the hospital (NS)	Yes	None	Adults with hypertension, diabetes, and/or cardiac arrhythmia
Greene, 2013	USA	Obesity	Accelerometer and weight scale connected to an online social network	Education on diet and physical activity	Weight (+)	Yes	Weight	Persons aged 17–79 who expressed concern about weight or health
Wang, 2015	USA	Obesity	Accelerometer with text messaging reminders	Self-monitoring with accelerometer only	Physical Activity (NS)	No	None	Non-smoking adults aged 18–69 who are overweight or obese (BMI ≥ 25)
Jakicic, 2016	USA	Obesity	Multi-sensor device worn on the upper arm provided feedback to the participant on energy expenditure and physical activity through a small display or through website	Diet, telephone counseling, group sessions, text message prompts, educational website	Weight (–)	No	Body mass index, weight, body fat percentage	Adults aged 18–35 with a body mass index between 25 and 40
Shuger, 2011	USA	Obesity	Physical activity monitor	Self-directed weight loss program	Body weight (+) and waist circumference (+)	No	Body mass index, weight, waist circumference, body fat percentage, systolic blood pressure	Underactive adults who are overweight or obese
Wijsman, 2013	Netherlands	Overweight	Accelerometer with personal website	Usual daily activity	Physical activity counts (+)	Yes	Body mass index, weight, waist circumference, body fat percentage, systolic blood pressure, diastolic blood pressure	Adults aged 60–70 years without diabetes
Kent, 2015	Australia	Sub-acute or chronic low back pain	Motion-sensor movement device with biofeedback	Motion-sensor without biofeedback	Self-reported pain intensity (+) and activity limitation (+)	Yes	None	Adults aged 18–65 presenting with a primary complaint of low back pain
Piga, 2014	Italy	Systemic sclerosis and rheumatoid arthritis	Telemonitoring system with hand exercises results transmitted to physicians	Standard at home kinesiotherapy exercises	Dreiser’s Index (NS), HAQ (NS), HAMIS hand (NS)	Yes	None	Adults diagnosed with systemic sclerosis or rheumatoid arthritis
Ryan, 2012	United Kingdom	Asthma	Spirometer with mobile application	Paper recording of peak flow and symptoms	Asthma control (NS), self efficacy (NS)	Yes	None	Adolescents and adults with poorly controlled asthma
Ginis, 2016	Belgium/Israel	Parkinson’s disease	Inertial measurement unit with smartphone application feedback	Weekly researcher visits without use of devices	Gait speed under usual and dual task conditions (NS)	No	None	Adults with Parkinson’s disease
Fox, 2012	Canada	Sleep apnea	PAP machine with transmission of physiologic information	Standard PAP machine	PAP adherence (+)	Yes	None	Adult patients with moderate-to-severe sleep apnea
Finkelstein, 2016	Singapore	No specific disease state	Sealed ActiGraph triaxial GT-3x + accelerometer and Fitbit Zip with website feedback	Education, cash incentives for participation	Moderate-to-vigorous physical activity per week (−)	No	Weight, systolic blood pressure	Full-time workers aged 21–65

*NS* not statistically significant, *COPD* chronic obstructive pulmonary disease, *HAQ* health assessment questionnaire, *HAMIS* hand mobility in scleroderma, *ECG* electrocardiogram, *GOLD* global initiative for chronic obstructive lung disease, *HRQL* health-related quality of life, *ED* emergency department, *PAP* positive airways pressure

#### Devices and interventions

RPM devices employed in these studies included blood pressure monitors, ambulatory electrocardiograms, cardiac event recorders, positive airway pressure machines, electronic weight scales, physical activity trackers and accelerometers, spirometers, and pulse oximeters. The control arms of most studies offered education along with standard care but without RPM; however, eight studies used other, similar devices. One study included various types of behavioral economics incentives (either donations to charity or cash incentives) in addition to the biosensors.^36^

Twenty-two study interventions contained a feedback loop with a care provider, such as a physician or nurse, who analyzed patient data and communicated back with the patient to modify treatment regimens, improve adherence, or consult. Only five study interventions contained a feedback loop where a care provider was not involved. In those instances, patients logged onto a web portal or mobile app to self-monitor their measurements and view a synthesis of their personal health data.

#### Qualitative review of high-quality studies

We examined the interventions, theoretical frameworks, and outcomes of the 16 high-quality studies by outcome or disease focus to determine if there were common intervention elements that resulted in greater effects on health and resource outcomes.

#### Remote patient monitoring for high-acuity patients: chronic obstructive pulmonary disease and heart failure

Five high-quality studies compared RPM with usual care for high-acuity patients with chronic obstructive pulmonary disease (COPD) or heart failure.^11,16,25,31,35^ In Chau et al., 40 participants with a previous hospitalization and diagnosed with moderate or severe COPD were randomized to usual care or a telecare device kit that provided patient feedback and was monitored by a community nurse.^11^ Although several participants experienced technical problems using the device kit, participants expressed greater engagement in self-management of their COPD overall. Nonetheless, the study found no positive effects in any of the primary outcomes when compared to usual care. As the authors note, the study was underpowered and had a short follow-up period of 2 months. In De San Miguel et al., the intervention was similar: 80 participants received telehealth equipment that monitored vital signs daily and was observed by a telehealth nurse.^25^ Patients in the intervention group experienced reductions in hospitalizations, emergency department visits, and length of stay, but none of the reductions were statistically significant when compared to the control group. Even so, the costs savings were $2931 per person, suggesting that a study with more power could potentially see significant cost and utilization savings. Dinesen et al. used a similar study design: 111 participants with COPD were randomized to receive telecare kits or usual care.^31^ The study found reductions in hospital admissions and lower costs of admissions in the intervention group, but only the mean hospital admission rate was statistically significant. Likewise, Pedone et al. followed 99 participants with COPD randomized to RPM or usual care, and found that the number of exacerbations and exacerbation-related hospitalizations dropped in the intervention group, but neither result was significant.^35^ The BEAT-HF trial by Ong et al. followed 1437 participants hospitalized with heart failure who were randomized to RPM or usual care.^16^ Centralized nurses actively monitored the RPM data. The researchers found no differences in 180-day all-cause readmissions between the two groups. Four studies demonstrate the promise of RPM for COPD-related hospitalizations and costs; longer follow-up periods and larger sample sizes are needed to determine the full effect of RPM on COPD outcomes. The use of measures such as the Patient Activation Measure^37^ in future studies could identify whether factors such as engagement and self-efficacy are important moderators of healthcare outcomes. More evidence is needed to determine whether heart failure is amenable to RPM; these patients may require more intensive follow-up care and may not be the ideal target population for RPM.

#### Remote patient monitoring for chronic disease: hypertension

Two high-quality studies focused on hypertension.^15,23^ Kim et al. examined 374 patients randomized to (1) home blood pressure monitoring, (2) remote monitoring using a wireless blood pressure cuff with clinician follow-up, or (3) remote monitoring without clinician follow-up.^23^ There were no differences observed in the primary endpoint, sitting systolic blood pressure, in the three groups. However, subjects over 55 years old with remote monitoring (with or without clinician follow-up) experienced significant decreases in the adjusted mean sitting systolic blood pressure when compared to the control group. These results indicate that for a select group of patients, RPM could be effective in hypertension treatment. Logan et al. provided home blood pressure telemonitoring with self-care messages on a smartphone after each reading for patients in the intervention group.^15^ Messages were tailored based on care pathways defined by running averages of blood pressure measurements. Physicians were alerted if patients’ blood pressure crossed specific pre-set thresholds and regular feedback was provided to patients and clinicians. Systolic blood pressure decreased in the intervention group; however, self-care smartphone-based support also appeared to worsen depression scores. These studies illustrate that tailored RPM interventions based on care pathways can effectively reduce blood pressure for select groups of patients, but researchers should examine adverse consequences such as depression and other patient-reported outcomes when designing interventions that include continuous monitoring.

#### Remote patient monitoring for rehabilitation: stroke, Parkinson’s disease, low back pain, and hand function

Four high-quality studies focused on providing feedback regarding various aspects of mobility rehabilitation, including stroke or Parkinson’s disease rehabilitation, low back pain physiotherapy, or hand function physical therapy.^20,24,27,28^ Dorsch et al. recruited 135 participants with stroke of any type from 16 rehabilitation centers in 11 countries.^24^ All participants wore wireless ankle tri-axial accelerometers while performing conventional rehabilitation exercises; intervention participants received and reviewed augmented feedback with therapists who used the wireless device data, while the control group received standardized verbal feedback from therapists. The researchers found no significant difference in the average daily time spent walking between groups throughout the duration of the trial. The researchers theorized that because participants walked such short amounts of time per day (a mean daily time of eight minutes in the severe group and 12 minutes in the moderate group), there was insufficient time to use the data provided by the wireless devices.

In Ginis et al., 40 participants with Parkinson’s disease undergoing gait training were randomized to home visits from the researcher who provided training on using a smartphone application and ankle-based wireless devices that offered positive and corrective feedback on gait, or an active control, in which they received personalized gait feedback from the same researcher during home visits.^20^ Both groups improved on the primary outcomes (single- and dual-task gait speed), but patients using the app and wireless devices improved significantly more on balance and experienced less deterioration over the six-week period.

Kent et al. randomized 112 participants in eight clinics between wearing active wireless motion sensors placed along the spine, and placebo sensors while receiving physical therapy and guideline-based care.^28^ Participants received six to eight physical therapy treatment sessions over 10 weeks and were followed for a year. Patients in the intervention group experienced significantly less pain and improved function compared to the control group. Designed as a pilot study, Piga et al. assigned 20 patients with systemic sclerosis or rheumatoid arthritis to use a self-managed hand kinesiotherapy protocol assisted by an RPM device.^27^ The device provides both visual and audio feedback on strength-, mobility-, and dexterity-based therapy. The control group received the kinesiotherapy protocol alone. Both groups improved over time, but there was no statistically significant difference in primary outcomes between the two groups. The researchers found, however, that measured adherence to the home-based RPM therapy was very high (90%). These four studies demonstrate mixed results on the use of RPM in rehabilitation but suggest potential insights. First, RPM is most useful in settings where there are clearly defined opportunities to use the data to change clinical care. For example, in the study examining stroke rehabilitation, participants did not walk enough throughout the day to effectively use the feedback. The study examining Parkinson’s disease rehabilitation, however, provided ample opportunities for participants to use the feedback over a six-week period, and participants saw important changes in clinical outcomes. Second, adherence to home-based rehabilitation therapy might be an important process outcome that could be included in future studies. Finally, using placebo sensors such as those used in Kent et al. is an important way to increase the validity and reliability of these studies.

#### Remote patient monitoring for increasing physical activity: overweightness and obesity

Five studies examined whether RPM could increase physical activity and combined activity monitors with a variety of behavioral interventions, including text messaging, personalized coaching, group-based behavior therapy, or cash- or charity-based incentives. In Wang et al., 67 participants who were overweight or obese were randomly assigned to wear a Fitbit One activity tracker alone or to wear the activity tracker combined with receiving physical activity prompts three times a day via text messages.^32^ The researchers found that both groups wearing the Fitbit devices saw a small increase in moderate-to-vigorous physical activity. Participants receiving the automatic text message prompts saw a small additional increase in activity that lasted only one week. Shuger et al. randomized 197 overweight or obese participants between four groups: (1) a control group that received a self-directed weight loss program via a manual, (2) a group that participated in a group-based behavioral weight loss program, (3) a group that received an armband (the SenseWear Armband) that monitored energy balance, daily energy expenditure, and energy intake, and (4) a group that received the armband and the group-based behavioral program.^19^ The group receiving the armband and group-based behavioral health intervention was the only one that achieved significant weight loss at nine months compared to the control group. Finkelstein et al. employed a behavioral economics study design, randomizing 800 participants from 13 companies in Singapore to one of four groups: (1) the control group, (2) Fitbit Zip activity tracker alone, (3) Fitbit Zip plus charity incentives, or (4) Fitbit Zip plus cash incentives.^36^ At 12 months, the Fitbit-only group and the Fitbit plus charity incentives group outperformed the control group and the Fitbit plus cash incentive group. The group receiving cash incentives saw a reduction in moderate-to-vigorous physical activity when compared to the control group.

Four hundred and seventy-one overweight and obese participants in Jakicic et al. received a low-calorie diet, prescription for physical activity, text message prompts, group counseling sessions, telephone counseling sessions, and access to materials on a website; the enhanced intervention group also received an activity tracker (FIT Core) that displayed data via the device interface or a website.^38^ The group that used the activity tracker experienced a lower amount of weight loss compared to the non-tracker group. Finally, in Wijsman et al., 235 participants aged 60–70 years without diabetes were randomized to the intervention group or a waitlist control group.^17^ Participants in the intervention group received a commercially available physical activity program (Philips DirectLife) based on the stages of change and I-change health behavior change models. The program includes an accelerometer-based activity tracker, a personal website, and a personal e-coach who provides support via email. After 13 weeks, daily physical activity increased significantly, and weight, waist circumference, and fat mass decreased significantly more in the intervention group compared to the control group. The results from these four different physical activity studies propose plausible directions into how and whether activity trackers can motivate behavior change. Cash incentives proved to be less effective than charity incentives, and automated, non-personalized text messages were also unproductive. Successful interventions combined RPM with several evidence-based components, including personalized coaching or group-based programs, or were grounded in validated behavior change models.

### Data analysis

For the meta-analysis, we created six groups of outcomes that had three or more studies, including: body mass index (BMI), weight, waist circumference, body fat percentage, systolic blood pressure, and diastolic blood pressure. There were no groupings found among the binary variables, so they were not included in the meta-analysis. In total, the meta-analysis included eight of the 27 studies.

### Body mass index (BMI)

**Fig. 2:**
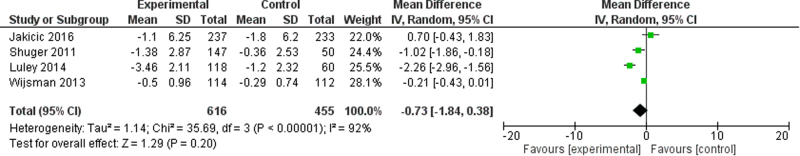
Point estimates of the mean difference for each study (green squares) and the corresponding 95% Confidence Intervals (horizontal black lines) are shown, with the size of the green square representing the relative weight of the study. The black diamond represents the overall pooled estimate, with the tips of the diamond representing the 95% Confidence Intervals

Four studies^17,19,33,38^ reported baseline and final outcome data for both intervention and control groups for BMI. The total aggregated calculation included 455 control patients and 616 intervention patients (Fig. 2). The meta-analysis yielded a mean difference point estimate of −0.73 (95% confidence interval: [−1.84, 0.38]), indicating no statistically significant difference between the experimental and control arms at the 95% confidence level with respect to whether RPM-based interventions resulted in a change in BMI. The I^2^ statistic was 92% (95% Confidence Interval: [83%, 96%]), illustrating a high degree of heterogeneity.

### Weight

**Fig. 3:**
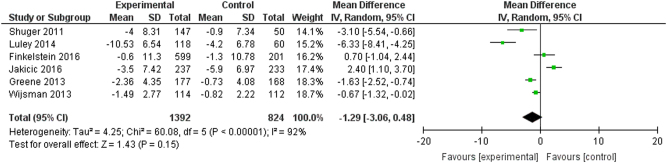
Point estimates of the mean difference for each study (green squares) and the corresponding 95% Confidence Intervals (horizontal lines) are shown, with the size of the green square representing the relative weight of the study. The black diamond represents the overall pooled estimate, with the tips of the diamond representing the 95% Confidence Intervals

Six studies^17,19,30,33,36,38^ reported data for both intervention and control groups for weight. The meta-analysis calculation was based on 824 control patients and 1392 intervention patients (Fig. 3). The meta-analysis yielded a mean difference point estimate of −1.29 (95% Confidence Interval: [−3.06, 0.48]), indicating no statistically significant difference. The I^2^ statistic was 92% (95% Confidence Interval: [85%, 96%]), illustrating a high degree of heterogeneity.

### Waist circumference

**Fig. 4:**
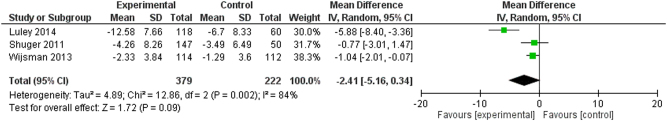
Point estimates of the mean difference for each study (green squares) and the corresponding 95% Confidence Intervals (horizontal lines) are shown, with the size of the green square representing the relative weight of the study. The black diamond represents the overall pooled estimate, with the tips of the diamond representing the 95% Confidence Intervals

Three studies^17,19,33^ reported data for both intervention and control groups for waist circumference, with a total of 222 control patients and 379 intervention patients **(**Fig. 4**)**. The meta-analysis yielded a mean difference point estimate of −2.41 (95% Confidence Interval: [−5.16, 0.34]), indicating no statistically significant difference. The I^2^ statistic was 84% (95% [51%, 95%]), illustrating a moderate to high degree of heterogeneity.

### Body fat percentage

**Fig. 5:**
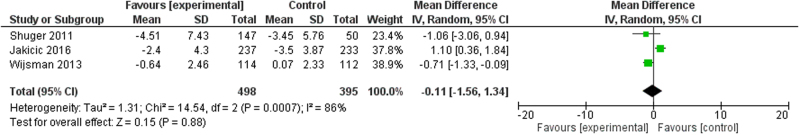
Point estimates of the mean difference for each study (green squares) and the corresponding 95% confidence intervals (horizontal lines) are shown, with the size of the green square representing the relative weight of the study. The black diamond represents the overall pooled estimate, with the tips of the diamond representing the 95% Confidence Intervals

Three studies^17,19,38^ reported data for both intervention and control groups for body fat percentage. There were a total of 395 control patients and 498 intervention patients **(**Fig. 5**)**. The meta-analysis yielded a mean difference point estimate of 0.11 (95% Confidence Interval: [−1.56, 1.34]), indicating no statistically significant difference. The I^2^ statistic was 86% (95% [59%, 95%]), illustrating a moderate to high degree of heterogeneity.

### Systolic blood pressure

**Fig. 6:**
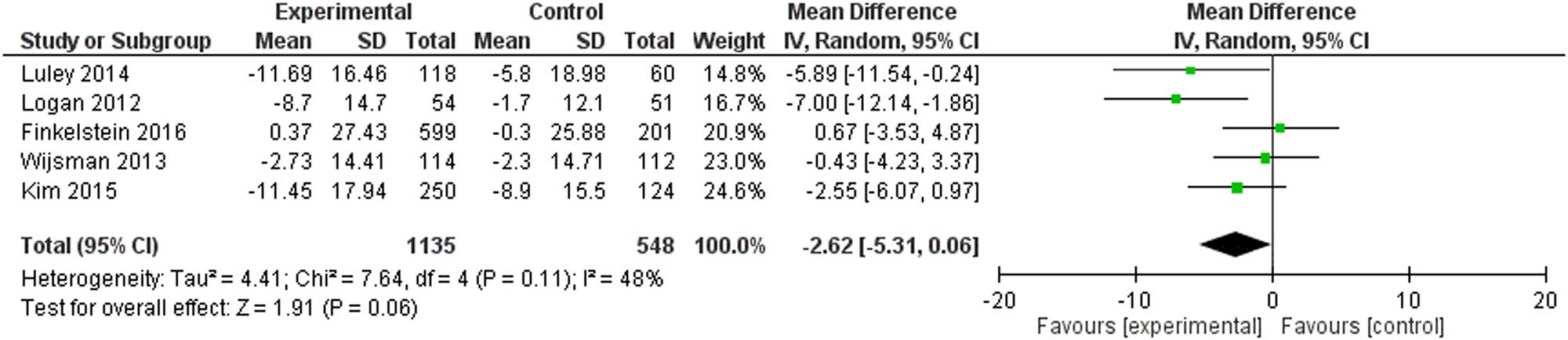
Point estimates of the mean difference for each study (green squares) and the corresponding 95% Confidence Intervals (horizontal lines) are shown, with the size of the green square representing the relative weight of the study. The black diamond represents the overall pooled estimate, with the tips of the diamond representing the 95% Confidence Intervals

Five studies^15,17,19,23,33,36^ reported data for both intervention and control groups for systolic blood pressure, with a total of 548 control patients and 1135 intervention patients (Fig. 6). The meta-analysis yielded a mean difference point estimate of −0.99 (95% Confidence Interval: [−2.73, 0.74]), indicating no statistically significant difference. The I^2^ statistic was 44% (95% [0%, 81%]), illustrating an unknown degree of heterogeneity.

### Diastolic blood pressure

**Fig. 7:**
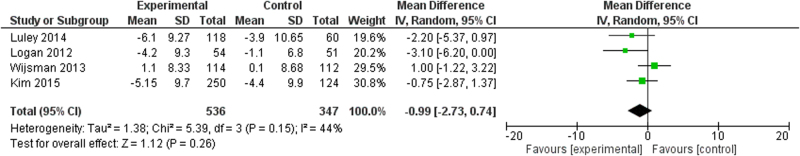
Point estimates of the mean difference for each study (green squares) and the corresponding 95% Confidence Intervals (horizontal lines) are shown, with the size of the green square representing the relative weight of the study. The black diamond represents the overall pooled estimate, with the tips of the diamond representing the 95% Confidence Intervals

Four studies^15,17,23,33^ reported data for both intervention and control groups for diastolic blood pressure, with a total of 347 control patients and 536 intervention patients (Fig. 7). The meta-analysis yielded a mean difference point estimate of −0.74 (95% Confidence Interval: [−2.34, 0.86]), indicating no statistically significant difference. The I^2^ statistic was 28% (95% [0%, 73%]), illustrating an unknown degree of heterogeneity.

## Discussion

Based on our systematic review and examination of high-quality studies on RPM, we found that remote patient monitoring showed early promise in improving outcomes for patients with select conditions, including obstructive pulmonary disease, Parkinson’s disease, hypertension, and low back pain. Interventions aimed at increasing physical activity and weight loss using various activity trackers showed mixed results: cash incentives and automated text messages were ineffective, whereas interventions based on validated health behavior models, care pathways, and tailored coaching were the most successful. However, even within these interventions, certain populations appeared to benefit more from RPM than others. For example, only adults over 55 years of age saw benefits from RPM in one hypertension study. Future studies should be powered to analyze sub-populations to better understand when and for whom RPM is most effective.

For the meta-analyses, we examined six different outcomes (BMI, weight, waist circumference, body fat percentage, systolic blood pressure, and diastolic blood pressure), and found no statistically significant differences between the use of RPM devices and controls with regard to any of these outcomes. However, we were limited by high heterogeneity and scarcity of high-quality studies. The high degree of heterogeneity is likely due to differences in the types of devices used, follow-up periods, and the types of controls in each study. In summary, our results indicate that while some RPM interventions may prove to be promising in changing clinical outcomes in the future, there are still large gaps in the evidence base. Of note, we found that many currently available consumer products have not yet been tested in RCTs with clinically meaningful outcomes. Although some consumer-facing digital health products may be effective for promoting behavior change, there is currently a dearth of evidence that these devices achieve health benefits; more research is needed in this field. Patients, clinicians, and health system leaders should proceed with caution before implementing and using RPM to reliably change clinical outcomes.

Future research should identify and remedy potential barriers to RPM effectiveness on clinical outcomes. For example, factorial design trials should evaluate variants of an RPM intervention in terms of frequency, duration, intensity, and timing. We also found that there are few large-scale clinical trials demonstrating a clinically meaningful impact on patient outcomes. Only one study identified in this review had a sample size of more than 1000 patients; most studies included fewer than 200 patients. Additionally, most studies had relatively short follow-up periods. Given that many of these studies were described as pilot studies, it is clear that the field of RPM is relatively new and evolving. Larger studies with multiple intervention groups will be able to better distinguish which components are most effective and whether behavior change can be sustained over time using RPM.

Future studies would also highly benefit from a mixed-methods approach in which both patients and clinicians are interviewed. Adding a qualitative component would give researchers insight into which RPM elements best engage and motivate patients, nurses, allied health workers, and physicians. Behavior change is complex; understanding how and if specific devices and device-related interventions and incentives motivate health behavior change is an important area that is still not well understood. For example, previous studies have found that most devices result in only short-term changes in behavior and motivation.^39^ Activity trackers have been found to change behavior for only approximately three to six months.^40^ Studies in this review found that cash incentives performed worse than charity incentives, illustrating that incentivizing individuals is complex and nuanced. Gaining a better understanding of how individuals interface with these health-related technologies will assist in developing evidence-based devices that have the potential to change behavior over longer periods of time.

One of the challenges of this review was the relatively broad survey into the effectiveness of RPM on clinical outcomes. This broad approach allowed us to examine the similarities among interventions targeted at different conditions, but also made it difficult to combine results among studies using different devices and associated interventions. Additional limitations of this study include the use of one primary database, PubMed, to identify articles. However, we examined review articles to identify potential studies that may be listed in other databases. Additionally, the study question focused solely on non-invasive wearable devices and excluded invasive devices such as glucose sensors, on which there have been many studies. The scope of this study included only RCTs with clinically meaningful outcomes. These rigorous search criteria excluded studies without controls or randomization. While non-randomized studies may nonetheless inform the field of RPM, given the risk of selection bias inherent in non-randomized trials, we determined it was optimal to restrict the inclusion criteria to RCTs in this meta-analysis of controlled trials.

An inherent shortcoming of most wearable device studies is difficulty in following double-blind procedures; the intervention arms necessarily include patient engagement or, at minimum, placement of the device on the patient’s body, which can be difficult to blind. Some studies have used devices that were turned off or were non-functional to reduce a potential placebo or Hawthorne effect,^41^ but given the data feedback loop integrated into many of these devices, it is extremely difficult to blind the provider receiving the data, which may impact results. Nonetheless, this shortcoming would tend to benefit the active intervention, making it more likely to show a difference in an unblinded study.

For RPM interventions to impact healthcare, they will need to impact outcomes that matter to patients. Examples include patient-reported health related quality of life (HRQOL), symptom severity, satisfaction with care, resource utilization, hospitalizations, readmissions, and survival. There is little data investigating the impact of RPM on these outcome measures. It may strengthen the interventions if they are developed directly in partnership with end-users—i.e. patients themselves. Further research might also emphasize how to personalize RPM interventions, as described by Joseph Kvedar and others.^42–44^ This approach seeks to optimize applications and sensors within a biopsychosocial framework.^44^ By using validated behavior-based models from the psychological and public health literature that integrate a variety of data from time of day to step counts, to the local weather, to levels of depression or anxiety, these tailored applications aim to generate contextually appropriate, highly tailored messages to patients at the right time and right place.^42–45^ This approach might combine the most successful elements of the effective interventions in this review, including personalized coaching and feedback, in a more cost-effective manner. Additionally, given the pronounced challenges in changing health-related behaviors, incorporating well-researched theoretical frameworks into interventions, such as the Health Belief Model,^46^ the Stages of Change Model,^47^ or Theory of Reasoned Action/Planned Behavior,^48^ may be ultimately more successful than merely improving the technical aspects of RPM.

## Methods

### Study identification

We performed a systematic review of PubMed from January 2000 to October 2016 to identify RCTs that assessed clinical outcomes related to the use of non-invasive wearable biosensors versus a control condition. The subject headings and key words incorporated into the search strategy included:

(“biosensing techniques”[MeSH Terms] OR “Remote sensing technology”[MeSH] OR “remote sensing”[text word] OR “On body sensor”[text word] OR Biosensor*[text word] OR “Wearable device”[text word] OR “Constant health monitoring”[text word] OR “Wireless technology”[text word] OR “wearable sensor”[text word] OR “wearable”[text word] OR “medical sensor”[text word] OR “Body Sensor”[text word] OR “Passive monitor”[text word] OR “wireless monitor”[text word] OR “monitoring device”[text word] OR “wireless sensor”[text word]) AND (hasabstract[text] OR English[lang]) AND (“Clinical Trial “[Publication Type] OR “Randomized Controlled Trial “[Publication Type] OR “randomized”[tiab] OR “placebo”[tiab] OR “therapy”[sh] OR randomly[tiab] OR trial[tiab] OR groups[tiab]) NOT (“animals”[MeSH] NOT “humans”[MeSH]).

After an initial review of our search yield, we added the following subject headings and key words:

(“Remote monitoring”[text word] OR “Remote patient monitoring”[text word] OR “self-monitoring”[text word] OR “self-tracking”[text word] OR “remote tracking”[text word] OR “home monitoring”[text word] OR “wireless monitoring”[text word] OR “online monitoring”[text word] OR “online tracking”[text word] OR “telemonitoring”[text word] OR “ambulatory monitoring”[text word]) AND (“e-health”[text word] OR “m-health”[text word] OR “mobile”[text word] OR “mobile health”[text word] OR “telehealth”[text word] OR “telemedicine”[text word] OR “digital health”[text word] OR “digital medicine”[text word] OR ((“smartphone”[MeSH Terms] OR “smartphone”[All Fields]) AND text[All Fields] AND word[All Fields]) OR “social network”[text word] OR “Web based”[text word] OR “online portal”[text word] OR “internet based”[text word] OR “cell phone”[text word] OR “mobile phone”[text word]).

Additionally, we consulted references from a previous systematic review.^10^

### Study selection and data extraction

We assessed all titles for relevance and rejected titles if they fulfilled pre-specified exclusion criteria (Table 3). Eight trained investigators independently screened titles in pairs of two. We calculated Fleiss’ Kappa, a measure of the degree of consistency between two or more raters to ensure high inter-rater reliability, and aimed for a kappa higher than 0.85.^49^ For studies identified in the second review process, a second independent review was performed. Differences regarding inclusion and exclusion criteria were resolved through consensus. We followed a similar method to review abstracts for all studies that passed the title screening stage, and included any study that met all of the abstract inclusion criteria (Table 3).

**Table 3:** Study Inclusion and Exclusion Criteria

Inclusion Criteria	Exclusion Criteria
[1] the study included a device on or touching the human body that [2] sensed a biometric measure related to the body itself; [3] the study contained a relevant control group; [4] the device automatically transmitted data to a web portal or app that could be accessed by the patient and/or care provider; [5] if a care provider had access to patient device data, they communicated back to the patient in regards to those data; and [6] the study measured a meaningful, clinically relevant health outcome.	[1] Studies in languages other than English, [2] studies not concerned with human subjects, [3] studies conducted with regards to implantable or invasive or ingestible or injectable devices, [4] studies on the cellular, biochemical or microscale and [5] studies primarily focused on the theory, design or proof of concept of the device.

### Data abstraction and data management

Each study was jointly abstracted for data by two reviewers and the results were entered into a standardized abstraction form. For each study, the reviewers extracted data about the targeted disease state, device type, control intervention, clinically relevant outcomes, type of feedback loop, descriptive information of subjects, and study design. For the analysis, we examined only continuous variables.

For continuous variables, we used a difference-in-differences model to assess relative change between the baseline measure and final measure for control and treatment groups. If a study did not provide baseline data, we emailed the respective authors and requested the data. If we did not receive a reply or the authors did not have baseline data, we excluded the study from this analysis.

We standardized all studies to provide the change from baseline mean and standard deviation for both the experimental and control arms. If a study reported only standard errors, *p*-values, or confidence intervals, we converted these to standard deviations (see Appendix). If a study did not provide a standard deviation or any of the three statistics mentioned above, we contacted the primary author, as explained above, and excluded the study from this analysis if they could not provide that information. Many of the identified studies used more than one experimental arm; we followed methods from Cochrane to combine the two groups into one larger group (see Appendix).^50^ We directionally corrected all signs and adjusted any differences in units of calculation (i.e. lbs vs. kg).

Given the heterogeneity of the interventions and outcomes, we grouped the outcome variables into separate groups for analysis (e.g. cholesterol, blood pressure). This process was jointly completed by two reviewers, with any disagreements discussed with a third-party arbiter.

### Statistical analyses

We used Review Manager (Review Manager [RevMan] Version 5.3. Copenhagen: The Nordic Cochrane Centre, The Cochrane Collaboration, 2014) to conduct a difference-in-differences random effects analysis. We used a difference-in-differences random effects analysis to help control for the many differences in the studies and to limit heterogeneity. We weighted the studies by sample size and used 95% confidence intervals around our point estimates. We also assessed for heterogeneity using the I^2^ statistic and calculated the 95% confidence intervals using the standard methods described by Higgins et al.^51^ We did not perform tests for funnel plot asymmetry to examine publication bias given that this type of analysis is not recommended for meta-analyses with fewer than 10 studies.^52^

### Strength of the body of evidence

We assigned a score for methodological quality by applying the Jadad scale,^53^ a commonly used instrument for measuring the quality of randomized controlled trials. The score awards points for appropriate randomization, presence of concealed allocation, adequacy of double blinding, appropriateness of blinding technique, and documentation of withdrawals and dropouts. The score ranges from 0 to 5, where a score of ≥3 denotes “high quality” based on the original validation studies. We measured inter-rater agreement for each step with a k statistic, and adopted a threshold of ≥0.7 as the definition for acceptable agreement. Disagreements were adjudicated by discussion and consensus between the two primary reviewers and a third-party arbiter.

### Data availability

The data used in this study was manually abstracted from the 27 studies identified in the systematic review. The meta-analysis used data from 8 of those 27 studies, which are referenced at the relevant points in the paper.
