# Author Correction: Impact of remote patient monitoring on clinical outcomes: an updated meta-analysis of randomized controlled trials

**DOI:** 10.1038/s41746-018-0027-3

**Published:** 2018-04-09

**Authors:** Benjamin Noah, Michelle S. Keller, Sasan Mosadeghi, Libby Stein, Sunny Johl, Sean Delshad, Vartan C. Tashjian, Daniel Lew, James T. Kwan, Alma Jusufagic, Brennan M. R. Spiegel

**Affiliations:** 1 Division of Health Services Research, Cedars-Sinai Medical Center, Los Angeles, CA USA; 2 Cedars-Sinai Center for Outcomes Research and Education (CS-CORE), Los Angeles, CA USA; 3 Department of Health Policy and Management, UCLA Fielding School of Public Health, Los Angeles, CA USA; 4 Department of Medicine, University of Arizona, College of Medicine Tucson, Tucson, AZ USA; 5 Cedars-Sinai Medical Center, Los Angeles, CA USA; 6 American Journal of Gastroenterology, Bethesda, USA

**Keywords:** Disease prevention, Weight management, Health services

**Correction to**: *npj Digital Medicine* **1**:2; 10.1038/s41746-017-0002-4, published online 15 January 2018

The original version of this Review Article contained calculation errors within the abstract:

“Body mass index (−0.96; 95% CI: −2.30, 0.37)” has been corrected to “Body mass index (−0.73; 95% CI: −1.84, 0.38)”, “BMI I^2^ statistic 92% (95% Confidence Interval: [82%, 96%]” was corrected to “BMI I^2^ statistic 92% (95% Confidence Interval: [83%, 96%]”, “body fat percentage (0.19; −1.2,1.57)” has been corrected to “body fat percentage (−0.11; −1.56, 1.34)”, I^2^ statistic 85% (95% [56%, 95%])” was corrected to “body fat “I^2^ statistic 86% (95% [59%, 95%])”, and “diastolic blood pressure (−0.74; −2.34, 0.86)” has been corrected to “diastolic blood pressure (−0.99; −2.73, 0.74)”, “diastolic blood pressure I^2^ statistic 28% (95% [0%, 73%])” has been changed to “diastolic blood pressure I^2^ statistic 44% (95% [0%, 81%])”. These corrections have been repeated in the results section and Figs. 2, 5 and 7.

**Fig. 2:**
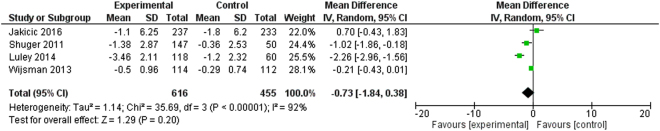
Point estimates of the mean difference for each study (green squares) and the corresponding 95% Confidence Intervals (horizontal black lines) are shown, with the size of the green square representing the relative weight of the study. The black diamond represents the overall pooled estimate, with the tips of the diamond representing the 95% Confidence Intervals

**Fig. 5:**
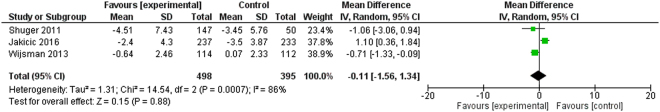
Point estimates of the mean difference for each study (green squares) and the corresponding 95% confidence intervals (horizontal lines) are shown, with the size of the green square representing the relative weight of the study. The black diamond represents the overall pooled estimate, with the tips of the diamond representing the 95% Confidence Intervals

**Fig. 7:**
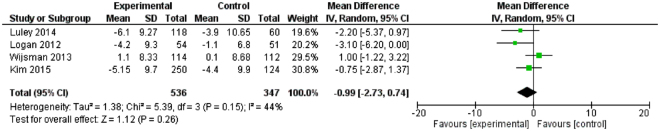
Point estimates of the mean difference for each study (green squares) and the corresponding 95% Confidence Intervals (horizontal lines) are shown, with the size of the green square representing the relative weight of the study. The black diamond represents the overall pooled estimate, with the tips of the diamond representing the 95% Confidence Intervals

These errors have now been corrected in the HTML and PDF versions of this Article.

